# Screening Immunoactive Compounds of *Ganoderma lucidum* Spores by Mass Spectrometry Molecular Networking Combined With *in vivo* Zebrafish Assays

**DOI:** 10.3389/fphar.2020.00287

**Published:** 2020-03-18

**Authors:** Zhenhao Li, Yingqiu Shi, Xiaohui Zhang, Jing Xu, Hanbo Wang, Lu Zhao, Yi Wang

**Affiliations:** ^1^Pharmaceutical Informatics Institute, College of Pharmaceutical Sciences, Zhejiang University, Hangzhou, China; ^2^Zhejiang Engineering Research Center of Rare Medicinal Plants, Hangzhou, China; ^3^Zhejiang Shouxiangu Institute of Rare Medicine Plant, Wuyi, China

**Keywords:** *Ganoderma lucidum* spore, mass spectrometry molecular networking, zebrafish-based bioassays, immunomodulatory effects, triterpenoids, partial least squares regression

## Abstract

*Ganoderma lucidum* is a well-known herbal remedy widely used for treating various chronic diseases. Traditionally, the fruiting body is regarded as the medicinal part of this fungus, while recently, the therapeutic potentials of *Ganoderma lucidum* spore (GLS) is gaining increasing interests. However, detailed knowledge of chemical compositions and biological activities of the spore is still lacking. In this study, high-resolution mass spectrometry and molecular networking were employed for in-depth chemical profiling of GLS, sporoderm-broken GLS (BGLS) and sporoderm-removed GLS (RGLS), leading to the characterization of 109 constituents. The result also showed that RGLS contained more triterpenoids with much higher contents than BGLS and GLS. Moreover, the immunomodulatory activities of BGLS and RGLS were investigated in the zebrafish models of neutropenia or macrophage deficiency. RGLS exhibited more potent activities in alleviating vinorelbine-induced neutropenia or macrophage deficiency, and significantly enhanced phagocytic function of macrophages, which indicated the immunomodulatory activity of GLS was positively correlated with the content of triterpenoids. Further correlation analysis of chemical profiles of GLS and corresponding bioactivities by partial least squares regression identified the potential immunoactive compounds of GLS, including 20-hydroxylganoderic acid G, elfvingic acid A and ganohainanic acid C. Our findings suggest that combining mass spectrometry molecular networking with zebrafish-based bioassays and chemometrics is a feasible strategy to reveal complex chemical compositions of herbal medicines, as well as to discover their potential active constituents.

## Introduction

*Ganoderma lucidum*, commonly known as Lingzhi or Reishi, has been used as an herbal remedy in China and many Asian countries for over 2000 years (Zhou et al., 2015; Yuan et al., 2018). According to traditional Chinese medicine (TCM) theory, *G. lucidum* can tonify “Qi,” and has been revered for its miracle cures and general health promoting benefits (Bishop et al., 2015). Modern scientific studies have proven that this medical macrofungus possesses various bioactivities, including immunomodulation, liver protection, diabetic treatment, anti-tumor and neuroprotective effects (Ahmad, 2018; Cao et al., 2018). Traditionally, the fruiting body of *G. lucidum* is used as the medicinal part and regarded as the source for many reported activities (Russell and Paterson, 2006; Hsu and Cheng, 2018). Less mature, but potentially even more valuable to therapeutic agent development, is the *Ganoderma lucidum* spore (GLS), the tiny reproduction unit of the fungus. Recently, GLS is gaining increasing acceptance and popularity as a functional food and nutraceutical, whose efficacy and safety have been suggested by multiple clinical studies in the treatment of cancers (Zhao et al., 2012; Hsu and Cheng, 2018), chronic periodontitis (Nayak et al., 2015) and Alzheimer disease (Wang et al., 2018). Although the use of GLS becomes popular, detailed knowledge of its chemical composition and biological activity is often lacking, as are data on the pharmacodynamics and clinical effects. Additionally, as GLS has outer bilayers of sporoderm, which is mainly composed of chitin and glucan (Lin and Wang, 2006), a variety of sporoderm-breaking techniques have been developed to release the components from the hard and resilient spores (Liu et al., 2005; Soccol et al., 2016). However, only a limited number of studies have been performed to investigate changes in chemical and biological properties of GLS after breaking the spore walls (Chen et al., 2012; Fu et al., 2012; Gao et al., 2013; Xu et al., 2014; Yang et al., 2017), and active constituents of GLS remain elusive (Liu et al., 2011; Yan et al., 2013).

Since its emergence, mass spectrometry (MS) is increasingly perceived as an essential tool in nearly all phases of drug discovery and development, including lead identification, metabolism, pharmacokinetics, and assessment of drug quality and safety (Hofstadler and Sannes-Lowery, 2006; Pacholarz et al., 2012). The hyphenated techniques, such as liquid chromatography-MS (LC-MS), and tandem MS (MS^2^), which represent the most widely used tools in MS arsenal, have shown many unique strengths in the drug discovery process. Recently, this cutting-edge technique has also been introduced into the realm of natural products and herbal medicines, which have been the source for new pharmaceutical drugs (Newman and Cragg, 2016). Different from synthetic or highly purified drugs, herbal medicines are complex mixtures, which usually contain hundreds of different phytochemicals. These herbal constituents generate thousands of molecular ions and fragment ions in MS analysis, rendering it challenging to annotate the detected chemical signatures. To address this issue, many MS data processing strategies have been developed to accelerate the dereplication and discovery process (Wang et al., 2016b, 2019; Li et al., 2017, 2019). Among these approaches, molecular networking (Watrous et al., 2012) is an emerging tool well suited to this task. Molecular networking visualizes all the ions and their chemical relationships based on MS^2^ spectra similarity, which is calculated by using a cosine score (Guthals et al., 2012; Quinn et al., 2017). In the generated molecular networks, each node represents a consensus MS^2^ spectrum (merged spectra with the same precursor ion and similar MS^2^ spectra), while the edges between the nodes indicate the degree of cosine similarity. Based on the established Global Natural Product Social Molecular Networking (GNPS, https://gnps.ucsd.edu) web platform (Wang et al., 2016a), researchers can elucidate the structure of analogs and structurally related molecules based on their MS spectra (Yang et al., 2013; Allard et al., 2016). Despite the great potential to aid dereplication and structure elucidation, the utility of molecular networking in herbal medicine discovery just begins (Ge et al., 2017; Li et al., 2018; Qiang et al., 2019), and improvements in method performance (e.g., in terms of algorithm, and bioactivity relevance) are still necessary.

Besides its applications in chemical identification, MS has been employed to screen active constituents from complex herbal medicines. Many strategies for active (constituents) identification based on MS have been proposed, which can be separated into two primary categories: the affinity ultrafiltration based strategies which directly detect target-ligand complexes or “free” ligands released from the noncovalent complexes (Chen et al., 2018); and the chemometrics based strategies which investigate the active constituents by correlating chemical profiles of herbal medicines to their bioactive effects (Chang et al., 2018). For example, Yang et al. developed an ultrafiltration high-performance liquid chromatography coupled with diode array detector and mass spectrometry (UF-HPLC-DAD-MS) method to screen tyrosinase inhibitors from mulberry leaves (Yang et al., 2012). Besides, in one of our previous studies, we explored the active constituents of a Chinese medicine (Wenxin Keli) by combining LC-MS, bioassays and an active index approaches (Liu et al., 2018). It is noteworthy that both types of methods own their distinctive pros and cons, and meticulous validation (e.g., *in silico* docking, dose-response tests, and *in vivo* pharmacological assays) is necessary when candidates are obtained.

**Figure 1:**
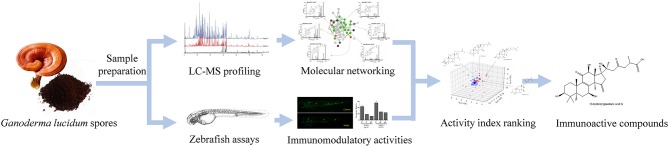
Scheme of identifying immunoactive compounds of *Ganoderma lucidum* spores by molecular networking, zebrafish assays and chemometrics.

In this work, the active constituents of GLS were investigated by correlating chemical profiles of GLS produced by different manufacturing processes to their respective *in vivo* activities using partial least squares regression. Molecular networking was employed for structure elucidation and in-depth chemical profiling of GLS. Moreover, the immunomodulatory activities of GLS were evaluated by the zebrafish models of neutrophil or macrophage deficiency, as well as the phagocytic capability of macrophages. The efficiency rate of each constituent was then calculated and ranked based on its peak areas in different GLS samples and the corresponding bioactivities. The experimental workflow is outlined schematically in Figure 1.

## Materials and Methods

### Materials and Reagents

*Ganoderma lucidum* spore (GLS, batch No. 14072201), sporoderm-broken *Ganoderma lucidum* spore (BGLS, batch No. 15121401) and sporoderm-removed *Ganoderma lucidum* spore (RGLS, batch No. 16042301) were provided by Zhejiang Shouxiangu Institute of Rare Medicine Plant (Zhejiang, China) and the species origin of the samples were authenticated as *Ganoderma lucidum* (Curtis) P. Karst. by Prof. Mingyan Li in the institute. The samples were kept in the sample room in Pharmaceutical Informatics Institute of Zhejiang University. Vinorelbine (batch No. 140501) was purchased from Jiangsu Haosen Pharmaceutical Co., Ltd (Jiangsu, China). Reference standards including ganoderic acid A, B, C_2_, D, DM, F, G, H, ganoderenic acid A, B, C, D, lucidenic acid A were purchased from Nature Standard (Shanghai, China).

HPLC-grade acetonitrile and methanol were purchased from Merck (Darmstadt, Germany). Formic acid (HPLC grade) was purchased from Roe Scientific (Newark, DE, USA). Ethanol and Methylcellulose were acquired from Shanghai Aladdin Bio-Chem Technology (Shanghai, China). Neutral red was purchased from Sigma Aldrich (Saint-Louis, MO, USA). Isopropyl alcohol was purchased from Hangzhou Changzheng Chemical Reagent (Hangzhou, China). Deionized water was prepared with an Elga PURELAB flex system (ELGA LabWater, UK).

### Sample Preparation

2 mg of GLS, BGLS, and RGLS were dissolved in 1 mL methanol respectively, and ultrasonically extracted for 20 min, and then centrifuged at 10,000 rpm for 10 min. The supernatants were collected for LC-MS analysis.

BGLS and RGLS were dissolved in system fish water at 10 mg/mL respectively, which were used for zebrafish assays. The system fish water was composed of 200 mg Instant Ocean Salt in per litter of reverse osmosis water with final pH 6.9–7.2, conductivity 480–510 μS/cm, and hardness of 53.7–71.6 mg/L CaCO_3_.

### Instrumentation

An Acquity UPLC system (Waters, Milford, MA, USA) coupled with a Triple TOF 5600plus MS (AB SCIEX, Framingham, MA, USA) was employed for chemical identification. Analysis was performed in negative electrospray ionization (ESI-) mode under following parameters: scan range, m/z 100–1500; source voltage, −4.5 kV; source temperature, 550°C; curtain gas, 35 psi; gas 1 (N_2_), 50 psi; gas 2 (N_2_), 50 psi. Declustering potential (DP), collision energy (CE) and collision energy spread (CES) of information dependent acquisition (IDA)-mediated MS^2^ were 100 V, 40 eV, and 20 eV, respectively.

Chromatographic separation was carried out on an Acquity BEH C18 column (100 mm × 2.1 mm, 1.7 μm, Waters) at 30°C with mobile phase A (0.1% v/v formic acid-water) and mobile phase B (acetonitrile). The flow rate was 0.4 mL/min and a linear gradient elution was programmed: 0–20 min, 20–40% B; 20–28 min, 40–50% B; 28–35 min, 50–70% B; 35–40 min, 70–80% B; 40–45 min, 80–90% B; 45–50 min, 90–95% B; 50–55 min, 95% B. The injection volume was 2 μL.

### Construction of MS/MS Based Molecular Network

Tandem mass spectrometry molecular networks were generated using the GNPS platform (https://gnps.ucsd.edu/). Raw MS data were first converted to mzXML format with MSConvert (Kessner et al., 2008) and then uploaded to GNPS to create the molecular networks. The precursor ion mass tolerance was set to 0.5 Da and to a product ion tolerance of 0.1 Da. A network was constructed using 6 minimum matched peaks and a cosine score above 0.7. The spectra in the network were searched against the spectral libraries on GNPS. Results were open and visualized in Cytoscape 3.7.1.

### Zebrafish Husbandry and Management

*Tg (mpx:GFP)* transgenic zebrafish that expressed GFP exclusively in neutrophils and *Albino* zebrafish were provided by Hunter Biotechnology, which is accredited by the International Association for Assessment and Accreditation of Laboratory Animal Care (AAALAC). Embryos were generated by natural pair-wise mating, and anesthetized in 0.016% (w/v) tricaine prior to observations.

### Zebrafish Model of Neutropenia

2-dpf *Tg (mpx:GFP)* transgenic zebrafish embryos were distributed into 6-well plates, with 30 larvae in 3 mL system fish water for each well. Three groups, i.e., the control group, the model group and the treatment group, were set up. Vinorelbine was administered at 1 ng per larva by intravenous microinjection to generate the zebrafish model of neutropenia. The treatment group was incubated in BGLS or RGLS supplemented fish water after microinjection. The final concentrations of BGLS (22, 67, and 200 μg/mL) and RGLS (33, 100, and 300 μg/mL) were set according to the maximum tolerated concentrations (MTCs) assay (Supplementary Methods). All the groups were incubated in a 28°C incubator for 24 h. Ten larvae were randomly selected from each group and the numbers of neutrophils in the zebrafish were counted and recorded with a Nikon Multi-purpose Zoom Microscope AZ100. The neutrophil recovery rate was calculated using the following formula:

$$\begin{array}{l} \text{neutrophil recovery rate}=\frac{N_{treatment}-N_{model}}{N_{model}}\times 100\% \end{array}$$

where *N*_*treatment*_ and *N*_*model*_ were the numbers of neutrophils of the larvae in the treatment group and model group, respectively.

### Zebrafish Model of Macrophage Deficiency

2-dpf *Albino* zebrafish embryos were distributed into 6-well plates, with 30 larvae in each well with 3 mL system fish water. Three groups were designed, including the control group, the model group and the treatment group. Vinorelbine was administered at 0.25 ng per larva by intravenous microinjection to generate the zebrafish model of macrophage deficiency. The treatment group was incubated in BGLS or RGLS supplemented fish water after microinjection. The final concentrations of BGLS (22, 67 and 200 μg/mL) and RGLS (111, 333 and 1000 μg/mL) were set according to the MTC assay in the *Albino* zebrafish (Supplementary Material). After 48 h incubation, the embryos were stained with 3 mL neutral red (2.5 μg/mL). Subsequently, the zebrafish embryos were immobilized in 3% methylcellulose, and the number of macrophages were counted and recorded with a Nikon dissecting microscope SMZ645. The macrophage formation efficiency was calculated using the following formula:

$$\begin{array}{l} \text{macrophage formation efficiency}=\frac{N_{treatment}-N_{model}}{N_{model}}\times 100\% \end{array}$$

where *N*_*treatment*_ and *N*_*model*_ were the numbers of macrophages of the larvae in the treatment group and model group, respectively.

### Zebrafish Model of Macrophage Phagocytosis

A zebrafish model of PM2.5 phagocytosis was used to assess the phagocytic function of macrophages under the effect of BGLS or RGLS. The model was created by injecting 10 nL active carbon nanoparticles (ACNP, 2.3 mg/mL) to 3-dpf *Albino* zebrafish. The group design and the tested concentrations were identical to the macrophage deficiency assay. After 24 h incubation, embryos were stained with 3 mL neutral red (2.5 μg/mL) and immobilized in 3% methylcellulose. The number of macrophages that phagocytized ACNP was then counted with a Nikon dissecting microscope SMZ645. The macrophage phagocytosis efficiency was calculated using the following formula:

$$\begin{array}{l} \text{macrophage phagocytosis efficiency}\\ =\frac{N_{treatment}-N_{model}}{N_{model}}\times 100\% \end{array}$$

where *N*_*treatment*_ and *N*_*model*_ were the numbers of macrophages that phagocytized ACNP in the treatment group and model group, respectively.

### Statistical Analysis

The data obtained from zebrafish assays was analyzed with GraphPad prism 7 software (GraphPad Software, USA). Parameter comparisons between groups were made with one-way ANOVA analysis of variance. The result was considered statistically significant when *P*-value < 0.05.

## Results and Discussion

### Molecular Networking to Profile *Ganoderma lucidum* Spores

**Figure 2:**
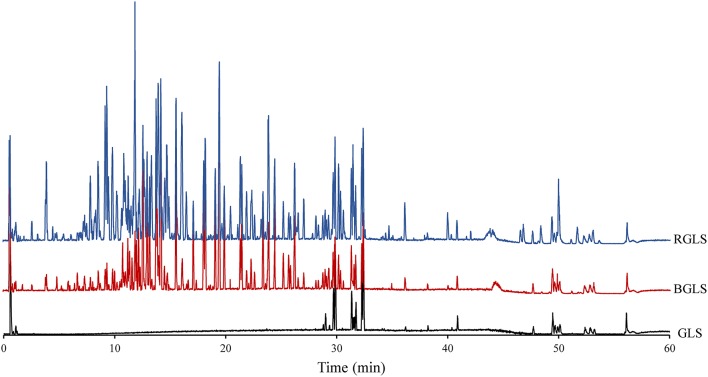
Representative base peak chromatograms of different *Ganoderma lucidum* spore samples obtained by LC-QTOF-MS in negative ion mode. BGLS, *Ganoderma lucidum* spore; BGLS, sporoderm-broken *Ganoderma lucidum* spore; RGLS, sporoderm-removed *Ganoderma lucidum* spore.

Representative UPLC–Q–TOF/MS chromatograms of GLS (raw material), sporoderm-broken *Ganoderma lucidum* spores (BGLS) and sporoderm-removed *Ganoderma lucidum* spores (RGLS) are shown in Figure 2. Apparently, the chemical profiles of these samples varied considerably. Only a few peaks were detected in the chromatogram of GLS, which suggested that the intact sporoderm acted as a barrier against the release of constituents inside the spores. In comparison, both BGLS and RGLS had much more peaks than GLS. The peak intensities of RGLS were higher than those of BGLS, which could be ascribed to the removal of the sporoderm in RGLS.

**Figure 3:**
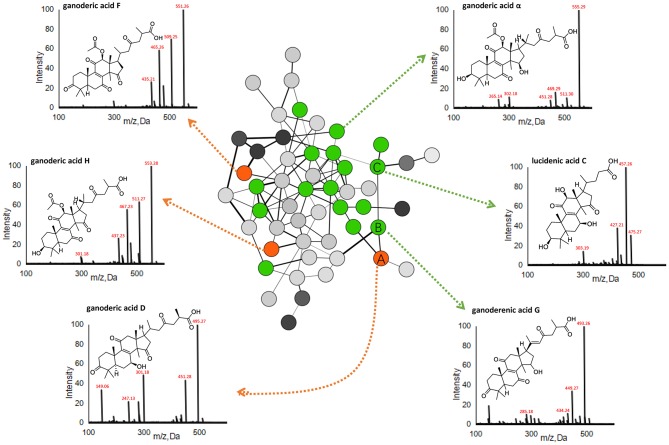
A representative MS/MS similarity network of the sporoderm-removed *Ganoderma lucidum* spore. Orange nodes represent reference triterpenoids (i.e., ganoderic acid D, H, and F), while green nodes represent identified triterpenoids.

Given the large quantity of constituents with diverse chemical structures in the spore, we next employed molecular networking for chemical identification, and focused on RGLS, which had the most peaks with higher intensities. The molecular network of RGLS based on MS^2^ spectra similarity was created on GNPS, which contained 501 distinguishable precursor ions, visualized as nodes in the network with 68 clusters (node ≥2), and 186 single nodes. Constituents were then identified and dereplicated through automatic searching in the spectral libraries on GNPS. Besides molecular networking, targeted LC-MS analysis was also conducted to assist the identification based on several strategies developed in our previous studies (Xiao et al., 2014; Li et al., 2015). Briefly, molecular formulae were first generated according to the high-resolution MS data, then the putative identification of the peaks was assigned based on literature and database matching, and was further confirmed via MS^2^ fragmentations. In addition, 13 constituents were unambiguously confirmed by comparisons with chemical standards in terms of retention time and mass spectra (Table 1). Nodes in the network corresponding to precursor ions of the 13 constituents were positioned and used to propagate molecular annotations, which accelerated dereplication of structurally related molecules. By applying these approaches, a total of 20, 96, and 109 constituents were identified or tentatively characterized from GLS, BGLS, and RGLS, respectively, including 99 triterpenoids, one linoleic acid, and 9 potentially new compounds (Table 1). Taking the cluster in Figure 3 as an example, node A showed a quasi-molecular ion [M-H]^−^ at *m/z* 513.2850, giving the formula C_30_H_42_O_7_. Fragment ions at *m/z* 495, 451, and 436 corresponded to successive losses of H_2_O, CO_2_, and CH_3_. Fragment ions at *m/z* 301 and 285 were characteristic ions formed by the cleavage of the D-ring ([M-H-C_11_H_16_O_4_]^−^) and the subsequent loss of CH_4_. In addition, this node showed identical retention time and similar mass spectra with those of the reference standard ganoderic acid D. Thus, this compound was unambiguously assigned as ganoderic acid D. Node B, which was adjacent to node A with high MS^2^ spectral similarity, showed 2 Da difference in the quasi-molecular ion and many fragment ions, indicating the two compounds shared similar structures. Moreover, node B exhibited identical fragments at *m/z* 301 and 285 with node A, which represented their A-, B-, and C-ring might be identical. Therefore, this compound was rapidly identified as ganoderenic acid G, with only one double-bond difference in the side chain. Likewise, as the neighbor of node B, node C exhibited a similar fragmentation pattern with node A and B, thus was identified as lucidenic acid C. Therefore, combining molecular networking with targeted MS analysis, large-scale MS dataset can be explored rapidly without any prior knowledge regarding the chemical compositions of the samples, and greatly facilitated the discovery of novel analogs. Detailed MS information is displayed in Table 1.

**Table 1:** Characterization of constituents in *Ganoderma lucidum* spore by LC-QTOF-MS.

**No**.	**RT (min)**	**Identity**	**Observed *m/z* (+/-)**	**Molecular formula**	**Error (ppm)**	**Major fragments**	**Source**
1	4.119	Lucidenic acid J	489.2485	C_27_H_38_O_8_	−1.8	489.2513 [M-H]^−^ 471.2390 [M-H-H_2_O]^−^ 459.2059 [M-H-2CH_3_]^−^ 441.1925 [M-H-CH_4_O-CH_3_]^−^ 346.1427 [M-H-CH_3_-C_7_H_12_O_2_]^−^ 318.1485 [M-H-C_8_H_12_O_3_-CH_3_]^−^	BGLS, RGLS
2	4.426	Elfvingic acid G	545.2745	C_30_H_42_O_9_	−2.0	545.2770 [M-H]^−^ 527.2667 [M-H-H_2_O]^−^ 497.2224 [M-H-H_2_O-2CH_3_]^−^ 483.2750 [M-H-H_2_O-CO_2_]^−^ 477.2869 [M-H-2H_2_O-2CH_4_]^−^ 453.2273 [M-H-H_2_O-CO_2_-2CH_3_]^−^	BGLS, RGLS
3	4.745	Lucidenic acid C	475.2688	C_27_H_40_O_7_	−2.8	475.2712 [M-H]^−^ 457.2608 [M-H-H_2_O]^−^ 265.1417 [M-H-C_12_H_18_O_3_]^−^	BGLS, RGLS
4	4.782	Ganoderic acid L	533.3109	C_30_H_46_O_8_	−2.0	533.3146 [M-H]^−^ 515.3015 [M-H-H_2_O]^−^ 317.1723 [M-H-C_10_H_16_O_4_]^−^ 303.1591 [M-H-C_11_H_18_O_5_]^−^	BGLS, RGLS
5	4.817	20-hydroxylganoderic acid G	547.2898	C_30_H_44_O_9_	−2.7	547.2974 [M-H]^−^ 529.2858 [M-H-H_2_O]^−^ 503.2624 [M-H-CO_2_]^−^ 485.2930 [M-H-H_2_O-CO_2_]^−^ 399.2180 [M-H-C_6_H_10_O_3_]^−^ 129.0507 [M-H-C_24_H_32_O_5_]^−^	BGLS, RGLS
6	4.983	Elfvingic acid D	545.2748	C_30_H_42_O_9_	−1.5	545.2757 [M-H]^−^ 527.2741 [M-H-H_2_O]^−^ 509.2525 [M-H-2H_2_O]^−^ 501.2896 [M-H-CO_2_]^−^ 415.2188 [M-H-C_6_H_10_O_3_]^−^ 129.0549 [M-H-C_24_H_32_O_6_]^−^	RGLS
7	5.184	Unknown	543.2593	C_30_H_40_O_9_	−1.2	543.2629 [M-H]^−^ 525.2499 [M-H-H_2_O]^−^ 499.2739 [M-H-CO_2_]^−^ 495.2378 [M-H-H_2_O-2CH_3_]^−^ 481.2601 [M-H-H_2_O-CO_2_]^−^ 455.2837 [M-H-C_3_H_4_O_3_]^−^ 437.2696 [M-H-C_3_H_4_O_3_-H_2_O]^−^ 407.2274 [M-H-C_3_H_4_O_3_-H_2_O-2CH_3_]^−^ 301.1789 [M-H-C_11_H_12_O_5_-H_2_O]^−^ 249.1469 [M-H-C_15_H_22_O_5_-H_2_O]^−^ 149.0944 [M-H-C_15_H_20_O_5_-C_6_H_12_O_2_]^−^	BGLS, RGLS
8	5.337	Elfvingic acid E	545.2748	C_30_H_42_O_9_	−1.5	545.2769 [M-H]^−^ 527.2691 [M-H-H_2_O]^−^ 497.2214 [M-H-H_2_O-2CH_3_]^−^ 483.2779 [M-H-H_2_O-CO_2_]^−^ 453.2277 [M-H-H_2_O-2CH_3_-CO_2_]^−^	BGLS, RGLS
9	5.417	3b,7b,12-Trihydroxy-4-(hydroxymethyl)-11,15,23-trioxolanost-8-en-26-oic acid	547.2904	C_30_H_44_O_9_	−1.6	547.2912 [M-H]^−^ 529.2827 [M-H-H_2_O]^−^ 511.2719 [M-H-2H_2_O]^−^ 485.2896 [M-H-H_2_O-CO_2_]^−^ 467.2808 [M-H-H_2_O-CO_2_-H_2_O]^−^ 265.1436 [M-H-C_15_H_22_O_5_]^−^	BGLS, RGLS
10	5.501	Ganoderic acid η	531.2946	C_30_H_44_O_8_	−3.3	531.2934 [M-H]^−^ 513.2875 [M-H-H_2_O]^−^ 469.2942 [M-H-H_2_O-CO_2_]^−^ 454.2739 [M-H-H_2_O-CO_2_-CH_3_]^−^ 319.1917 [M-H-C_11_H_16_O_4_]^−^ 301.1787 [M-H-C_11_H_16_O_4_-H_2_O]^−^ 265.1427 [M-H-C_15_H_22_O_4_]^−^	BGLS, RGLS
11	5.975	20-hydroxylucidenic acid F	471.2377	C_27_H_36_O_7_	−2.4	471.2372 [M-H]^−^ 441.2320 [M-H-2CH_3_]^−^ 427.2480 [M-H-CO_2_]^−^ 409.2368 [M-H-CO_2_-H_2_O]^−^ 367.2317 [M-H-C_3_H_6_O_2_-2CH_3_]^−^ 337.2166 [M-H-C_3_H_6_O_2_-4CH_3_]^−^	BGLS, RGLS
12	6.136	Ganoderic acid I	531.2952	C_30_H_44_O_8_	−2.2	531.2970 [M-H]^−^ 513.2873 [M-H-H_2_O]^−^ 483.2382 [M-H-H_2_O-2CH_3_]^−^ 401.2298 [M-H-C_6_H_10_O_3_]^−^ 129.0556 [M-H-C_24_H_34_O_5_]^−^	BGLS, RGLS
13	6.189	Lucidenic acid G	475.2685	C_27_H_40_O_7_	−3.4	475.2707 [M-H]^−^ 457.2609 [M-H-H_2_O]^−^ 439.2748 [M-H-2H_2_O]^−^ 427.2132 [M-H-H_2_O-2CH_3_]^−^ 409.2020 [M-H-3H_2_O-CH_3_]^−^ 385.2380 [M-H-C_2_H_4_O_2_-H_2_O-CH_3_]^−^ 303.1952 [M-H-C_8_H_10_O_3_-H_2_O]^−^ 287.1650 [M-H-C_8_H_10_O_3_-H_2_O-CH_3_]^−^ 285.1861 [M-H-C_8_H_12_O_3_-H_2_O-CH_3_]^−^	BGLS, RGLS
14	6.75	Methyl lucidenate E2	515.3005	C_30_H_44_O_7_	−1.8	515.3032 [M-H]^−^ 453.3007 [M-H-C_2_H_2_O]^−^ 441.2659 [M-H-C_3_H_6_O_2_]^−^ 426.2421 [M-H-C_3_H_6_O_2_-CH_3_]^−^ 407.2220 [M-H-C_3_H_6_O_2_-CH_4_-H_2_O]^−^ 303.2000 [M-H-C_11_H_16_O_4_]^−^ 249.1502 [M-H-C_15_H_22_O_4_]^−^ 137.0622 [M-H-C_21_H_30_O_6_]^−^ 73.0301 [M-H-C_26_H_34_O_6_]^−^	RGLS
15	6.956	20-hydroxylucidinic acid A	473.2536	C_27_H_38_O_7_	−1.9	473.2559 [M-H]^−^ 443.2080 [M-H-2CH_3_]^−^ 302.1497 [M-H-C_8_H_12_O_4_-H]^−^	BGLS, RGLS
16	7.054	Lucidenic acid M	461.2895	C_27_H_42_O_6_	−3.0	461.2917 [M-H]^−^ 417.3016 [M-H-CO_2_]^−^ 302.1915 [M-H-C_8_H_14_O_3_-H]^−^ 301.1775 [M-H-C_8_H_16_O_3_]^−^ 287.1648 [M-H-C_8_H_16_O_3_-CH_3_]^−^	BGLS, RGLS
17^a^	7.243	Ganoderenic acid C	515.3005	C_30_H_44_O_7_	−2.2	515.3050 [M-H]^−^ 303.1968 [M-H-C_11_H_16_O_4_]^−^ 211.0985 [M-H-C_19_H_28_O_3_]^−^ 193.0863 [M-H-C_19_H_28_O_3_-H_2_O]^−^	RGLS
18	7.287	Elfvingic acid H	529.2798	C_30_H_42_O_8_	−1.7	529.2836 [M-H]^−^ 511.2720 [M-H-H_2_O]^−^ 481.2263 [M-H-H_2_O-2CH_3_]^−^ 467.2814 [M-H-H_2_O-CO_2_]^−^ 437.2330 [M-H-H_2_O-CO_2_-2CH_3_]^−^ 303.1594 [M-H-C_11_H_14_O_5_]^−^ 261.1488 [M-H-C_15_H_24_O_4_]^−^	BGLS, RGLS
19	7.539	Lucidenic acid C	475.2690	C_27_H_40_O_7_	−2.4	475.2698 [M-H]^−^ 457.2593 [M-H-H_2_O]^−^ 439.2490 [M-H-2H_2_O]^−^ 427.2140 [M-H-H_2_O-2CH_3_]^−^ 409.2018 [M-H-3H_2_O-CH_3_]^−^ 303.2026 [M-H-C_8_H_10_O_3_-H_2_O]^−^ 287.1619 [M-H-C_8_H_10_O_3_-H_2_O-CH_3_]^−^	BGLS, RGLS
20	7.858	Butyl lucidenate E2	571.2908	C_32_H_44_O_9_	−0.8	571.2950 [M-H]^−^ 529.2833 [M-H-C_2_H_2_O]^−^ 511.2745 [M-H-C_2_H_4_O_2_]^−^ 499.2366 [M-H-C_2_H_2_O-2CH_3_]^−^ 497.2579 [M-H-C_2_H_2_O-2CH_4_]^−^	BGLS, RGLS, GLS
						467.2823 [M-H-C_2_H_4_O_2_-CO_2_]^−^ 455.2459 [M-H-C_2_H_4_O_2_-CO_2_-H_2_O]^−^ 440.2232 [M-H-C_2_H_4_O_2_-CO_2_-H_2_O-CH_3_]^−^ 425.1991 [M-H-C_2_H_4_O_2_-CO_2_-H_2_O-2CH_3_]^−^	
21^a^	7.868	Ganoderic acids C2	517.3165	C_30_H_46_O_7_	−1.1	517.3205 [M-H]^−^ 499.3087 [M-H-H_2_O]^−^ 455.3166 [M-H-H_2_O-CO_2_]^−^ 437.3088 [M-H-H_2_O-CO_2_-H_2_O]^−^ 303.1961 [M-H-C_11_H_18_O_4_]^−^ 301.1806 [M-H-C_11_H_20_O_4_]^−^ 287.1646 [M-H-C_11_H_18_O_4_-CH_4_]^−^ 249.1494 [M-H-C_15_H_24_O_4_]^−^	BGLS, RGLS, GLS
22	7.964	Elfvingic acid B	527.2642	C_30_H_40_O_8_	−1.6	527.2662 [M-H]^−^ 509.2569 [M-H-H_2_O]^−^	BGLS, RGLS
						479.2095 [M-H-H_2_O-2CH_3_]^−^ 465.2664 [M-H-H_2_O-CO_2_]^−^ 435.2190 [M-H-H_2_O-CO_2_-2CH_3_]^−^ 330.1473 [M-H-C_10_H_16_O_4_-H]^−^	
23	8.229	iso-ganoderic acid G	531.2953	C_30_H_44_O_8_	−2.0	531.3005 [M-H]^−^ 513.2891 [M-H-H_2_O]^−^ 469.2979 [M-H-H_2_O-CO_2_]^−^ 451.2869 [M-H-2H_2_O-CO_2_]^−^ 303.1953 [M-H-C_11_H_16_O_5_]^−^ 287.1639 [M-H-C_11_H_16_O_5_-CH_3_]^−^ 265.1443 [M-H-C_15_H_22_O_4_]^−^	BGLS, RGLS
24	8.268	Ganoderic acid θ	529.2800	C_30_H_42_O_8_	−1.3	511.2724 [M-H-H_2_O]^−^ 481.2258 [M-H-H_2_O-2CH_3_]^−^ 467.2823 [M-H-H_2_O-CO_2_]^−^ 449.2697 [M-H-H_2_O-CO_2_-H_2_O]^−^ 437.2347 [M-H-H_2_O-2CH_3_-CO_2_]^−^ 303.1588 [M-H-C_11_H_14_O_5_]^−^	BGLS, RGLS
25	8.268	Elfvingic acid F	545.2751	C_30_H_42_O_9_	−0.9	545.2775 [M-H]^−^ 527.2713 [M-H-H_2_O]^−^ 415.2153 [M-H-C_6_H_10_O_3_]^−^ 397.2028 [M-H-C_11_H_18_O_5_-H_2_O]^−^ 283.1693 [M-H-C_16_H_16_O_5_-H_2_O-CH_4_]^−^ 129.0546 [M-H-C_24_H_32_O_6_]^−^	BGLS, RGLS
26	8.349	Lucidenic acid N	459.2745	C_27_H_40_O_6_	−1.6	459.2772 [M-H]^−^ 441.2644 [M-H-H_2_O]^−^ 287.2004 [M-H-C_8_H_12_O_3_-CH_3_]^−^ 249.1482 [M-H-C_12_H_18_O_3_]^−^ 209.1176 [M-H-C_11_H_22_O_3_]^−^	BGLS, RGLS
27	8.575	Ganoderic acid C_6_	529.2800	C_30_H_42_O_8_	−1.3	511.2734 [M-H-H_2_O]^−^ 481.2264 [M-H-H_2_O-2CH_3_]^−^ 467.2832 [M-H-H_2_O-CO_2_]^−^ 437.2357 [M-H-H_2_O-CO_2_-2CH_3_]^−^ 303.1593 [M-H-H-C_11_H_14_O_5_]^−^	BGLS, RGLS, GLS
28	8.814	Dehydrolucidenic acid N	457.2589	C_27_H_38_O_6_	−1.4	457.2603 [M-H]^−^ 413.2696 [M-H-CO_2_]^−^ 397.2389 [M-H-CO_2_-CH_3_]^−^ 385.2388 [M-H-CO_2_-2CH_3_]^−^ 353.2489 [M-H-CO_2_-2CH_3_-2H_2_O]^−^ 285.1824 [M-H-C_8_H_10_O_3_-H_2_O]^−^ 249.1475 [M-H-C_12_H_16_O_3_]^−^	BGLS, RGLS
29	8.876	Elfvingic acid A	527.2644	C_30_H_40_O_8_	−1.2	527.2660 [M-H]^−^ 509.2572 [M-H-H_2_O]^−^ 497.2231 [M-H-2CH_3_]^−^ 479.2083 [M-H-H_2_O-2CH_3_]^−^ 465.2688 [M-H-H_2_O-CO_2_]^−^ 453.2293 [M-H-2CH_3_-CO_2_]^−^ 435.2181 [M-H-2CH_3_-H_2_O-CO_2_]^−^	BGLS, RGLS
30^a^	9.199	Ganoderic acid G	531.2955	C_30_H_44_O_8_	−1.6	513.2876 [M-H-H_2_O]^−^ 469.2993 [M-H-H_2_O-CO_2_]^−^ 451.2874 [M-H-2H_2_O-CO_2_]^−^ 436.2661 [M-H-2H_2_O-CO_2_-CH_3_]^−^ 303.1973 [M-H-C_11_H_16_O_5_]^−^ 287.1635 [M-H-C_11_H_16_O_5_-CH_3_]^−^ 265.1427 [M-H-C_15_H_22_O_4_]^−^ 249.1474 [M-H-C_15_H_22_O_5_]^−^	BGLS, RGLS, GLS
31	9.225	Elfvingic acid C	529.2797	C_30_H_42_O_8_	−1.9	529.2873 [M-H]^−^ 511.2732 [M-H-H_2_O]^−^ 467.2840 [M-H-H_2_O-CO_2_]^−^ 449.2721 [M-H-H_2_O-CO_2_-H_2_O]^−^ 318.1837 [M-H-C_10_H_14_O_4_-CH_3_]^−^ 301.1832 [M-H-C_11_H_16_O_5_]-	BGLS, RGLS
32	9.272	Lucidenic acid P	517.2799	C_29_H_42_O_8_	−1.5	517.2826 [M-H]^−^ 499.2721 [M-H-H_2_O]^−^ 475.2706 [M-H-C_2_H_2_O]^−^ 457.2609 [M-H-C_2_H_4_O_2_]^−^ 439.2506 [M-H-C_2_H_4_O_2_-H_2_O]^−^ 427.2131 [M-H-C_2_H_4_O_2_-2CH_3_]^−^ 409.2024 [M-H-C_2_H_4_O_2_-2H_2_O-CH_3_]^−^ 303.1987 [M-H-C_2_H_4_O_2_-C_8_H_10_O_3_]^−^	BGLS, RGLS
33^a^	9.504	Ganoderenic acid B	513.2852	C_30_H_42_O_7_	−1.1	559.2908 [M-H+FA]^−^ 513.2882 [M-H]^−^ 495.2767 [M-H-H_2_O]^−^ 451.2869 [M-H-H_2_O-CO_2_]^−^ 436.2628 [M-H-H_2_O-CO_2_-CH_3_]^−^ 421.2393 [M-H-H_2_O-CO_2_-2CH_3_]^−^ 331.1912 [M-H-C_10_H_14_O_3_]^−^ 303.1964 [M-H-C_11_H_14_O_4_]^−^ 249.1491 [M-H-C_15_H_20_O_4_]^−^	BGLS, RGLS
34	9.582	Lucidenic acid I	473.2532	C_27_H_38_O_7_	−2.7	473.2572 [M-H]^−^ 455.2459 [M-H-H_2_O]^−^ 437.2368 [M-H-2H_2_O]^−^ 425.1966 [M-H-H_2_O-2CH_3_]^−^ 301.1790 [M-H-C_8_H_10_O_3_-H_2_O]^−^ 285.1484 [M-H-C_8_H_10_O_3_-H_2_O-CH_3_]^−^	BGLS, RGLS
35	9.81	12-deacetylganoderic acid H	529.2803	C_30_H_42_O_8_	−0.7	529.2833 [M-H]^−^ 511.2734 [M-H-H_2_O]^−^ 493.2637 [M-H-2H_2_O]^−^ 467.2820 [M-H-H_2_O-CO_2_]^−^ 301.1808 [M-H-C_11_H_16_O_5_]^−^	BGLS, RGLS
36	9.854	ganoderic acid GS-1	497.2904	C_30_H_42_O_6_	−1.4	527.2672 [M-H]^−^ 509.2561 [M-H-H_2_O]^−^ 491.2453 [M-H-2H_2_O]^−^ 465.2645 [M-H-H_2_O-CO_2_]^−^ 447.2541 [M-H-2H_2_O-CO_2_]^−^ 301.1801 [M-H-C_11_H_14_O_5_]^−^ 299.1644 [M-H-C_11_H_16_O_5_]^−^	BGLS, RGLS
37	9.858	Ganoderic acid ε or ganoderic acid δ	515.3009	C_30_H_44_O_7_	−1.0	561.3045 [M-H+FA]^−^ 515.3040 [M-H]^−^ 497.2931 [M-H-H_2_O]^−^ 471.3157 [M-H-CO_2_]^−^ 341.2115 [M-H-C_8_H_12_O_3_-H_2_O]^−^	BGLS, RGLS, GLS
38	10.235	Unknown	513.2851	C_30_H_42_O_7_	−1.3	513.2897 [M-H]^−^ 495.2782 [M-H-H_2_O]^−^ 451.2871 [M-H-H_2_O-CO_2_]^−^ 436.2629 [M-H-H_2_O-CO_2_-CH_3_]^−^ 301.1796 [M-H-C_11_H_16_O_4_]^−^ 249.1476 [M-H-C_15_H_20_O_4_]^−^	BGLS, RGLS
39	10.287	Lucidenic acid E2	515.2648	C_29_H_40_O_8_	−0.5	515.2701 [M-H]^−^ 473.2575 [M-H-C_2_H_2_O]^−^ 443.2099 [M-H-C_3_H_4_O_2_]^−^	BGLS, RGLS, GLS
40	10.679	12β-acetoxy-7β-hydroxy-3,11,15,23-tetraoxo-5α-lanost-8-en-26-oic acid	571.2911	C_32_H_44_O_9_	−0.3	571.2943 [M-H]^−^ 553.2838 [M-H-H_2_O]^−^ 511.2734 [M-H-C_2_H_4_O_2_]^−^ 467.2835 [M-H-C_2_H_4_O_2_-CO_2_]^−^ 449.2720 [M-H-C_2_H_2_O_2_-CO_2_-H_2_O]^−^ 303.1973 [M-H-C_11_H_12_O_4_-C_2_H_4_O_2_]^−^	BGLS, RGLS, GLS
41	10.683	Lucidenic acid K	471.2382	C_27_H_36_O_7_	−1.3	471.2398 [M-H]^−^ 453.2310 [M-H-H_2_O]^−^ 441.1926 [M-H-2CH_3_]^−^ 300.1348 [M-H-C_8_H_10_O_4_-H]^−^	BGLS, RGLS
42	10.875	Ganoderic acid α	573.3061	C_32_H_46_O_9_	−1.4	573.3061 [M-H]^−^ 555.2990 [M-H-H_2_O]^−^ 511.3083 [M-H-H_2_O-CO_2_]^−^ 469.2987 [M-H-C_4_H_6_O_2_-H_2_O]^−^ 451.2862 [M-H_2_O-CO_2_-C_2_H_4_O_2_]^−^ 265.1439 [M-H-C_17_H_24_O_5_]^−^	BGLS, RGLS
43	10.972	Lucidenic acid B	473.2534	C_27_H_38_O_7_	−2.3	473.2566 [M-H]^−^ 455.2454 [M-H-H_2_O]^−^ 437.2338 [M-H-2H_2_O]^−^ 425.1965 [M-H-H_2_O-2CH_3_]^−^ 422.2109 [M-H-2H_2_O-CH_3_]^−^ 407.1881 [M-H-2H_2_O-2CH_3_]^−^ 301.1806 [M-H-C8H10O3-H2O]^−^	BGLS, RGLS
44	11.025	Ganoderic acid V1	513.2850	C_30_H_42_O_7_	−1.5	513.2883 [M-H]^−^ 495.2767 [M-H-H_2_O]^−^ 301.1822 [M-H-C_11_H_16_O_4_]^−^	BGLS, RGLS
						285.1513 [M-H-C_11_H_14_O_4_-H_2_O]^−^ 193.0865 [M-H-C_19_H_26_O_3_-H_2_O]^−^	
45	11.139	Applanoxidic acid G	527.2642	C_30_H_40_O_8_	−1.6	527.2696 [M-H]^−^ 509.2568 [M-H-H_2_O]^−^ 479.2112 [M-H-H_2_O-2CH_3_]^−^ 465.2675 [M-H-H_2_O-CO_2_]^−^ 435.2198 [M-H-H_2_O-CO_2_-2CH_3_]^−^ 301.1454 [M-H-C_11_H_14_O_5_]^−^ 299.1658 [M-H-C_11_H_16_O_5_]^−^	BGLS, RGLS
46	11.287	Lucidenic acid Q	459.2743	C_27_H_40_O_6_	−2.0	505.2799 [M-H+FA]^−^ 441.2610 [M-H-H_2_O]^−^ 397.2714 [M-H-H_2_O-CO_2_]^−^ 299.1634 [M-H-C8H16O3]^−^ 285.1477 [M-H-C8H16O3-H2O]^−^	BGLS, RGLS
47	11.529	Ganoderic acid N	529.2800	C_30_H_42_O_8_	−1.3	529.2854 [M-H]^−^ 511.2741 [M-H-H_2_O]^−^ 467.2831 [M-H-H_2_O-CO_2_]^−^ 449.2744 [M-H-2H_2_O-CO_2_]^−^ 285.1492 [M-H-C_11_H_14_O_4_-H_2_O-CH_4_]^−^ 263.1283 [M-H-C_15_H_22_O_4_]^−^	BGLS, RGLS
48	11.549	Applanoxidic acid C	525.2484	C_30_H_38_O_8_	−1.9	525.2543 [M-H]^−^ 507.2411 [M-H-H_2_O]^−^ 477.1950 [M-H-H_2_O-2CH_3_]^−^ 463.2493 [M-H-H_2_O-CO_2_]^−^ 433.2030 [M-H-H_2_O-2CH_3_-CO_2_]^−^ 328.1312 [M-H-C_10_H_13_O_4_]^−^ 299.1631 [M-H-C_11_H_14_O_5_]^−^	BGLS, RGLS
49	11.558	3β-hydroxy-12β-acetoxyganodernoid D	569.2751	C_32_H_42_O_9_	−0.9	569.2776 [M-H]^−^ 551.2675 [M-H-H_2_O]^−^ 509.2562 [M-H-H_2_O-C_2_H_2_O]^−^ 479.2092 [M-H-H_2_O-C_2_H_2_O-2CH_3_]^−^	BGLS, RGLS
						465.2648 [M-H-H_2_O-C_2_H_2_O-CO_2_]^−^ 435.2174 [M-H-H_2_O-C_2_H_2_O-CO_2_-2CH_3_]^−^ 345.1687 [M-H-C_11_H_14_O_4_-CH_3_]^−^ 330.1467 [M-H-C_11_H_14_O_4_-2CH_3_]^−^ 301.1784 [M-H-C_11_H_12_O_4_-C_2_H_4_O_2_]^−^	
50	11.611	Ganoderic acid O	527.2643	C_30_H_40_O_8_	−1.4	527.2672 [M-H]^−^ 509.2561 [M-H-H_2_O]^−^ 491.2453 [M-H-2H_2_O]^−^ 465.2645 [M-H-H_2_O-CO_2_]^−^ 447.2541 [M-H-2H_2_O-CO_2_]^−^ 301.1801 [M-H-C_11_H_14_O_5_]^−^ 299.1644 [M-H-C_11_H_16_O_5_]^−^	BGLS, RGLS
51^a^	11.827	Ganoderic acid H	571.2909	C_32_H_44_O_9_	−0.6	553.2828 [M-H-H_2_O]^−^ 511.2720 [M-H-C_2_H_4_O_2_]^−^ 509.2928 [M-H-H_2_O-CO_2_]^−^ 481.2244 [M-H-C_2_H_4_O_2_-2CH_3_]^−^ 467.2342 [M-H-H_2_O-CO_2_-C_2_H_2_O_2_]^−^ 437.2342 [M-H-H_2_O-CO_2_-C_2_H_2_O_2_-2CH_3_]^−^ 449.2701 [M-H-C_2_H_4_O_2_-H_2_O-CO_2_]^−^ 303.1592 [M-H-C_11_H_12_O_4_-C_2_H_4_O_2_]^−^ 301.1803 [M-H-C_11_H_14_O_4_-C_2_H_4_O_2_]^−^	BGLS, RGLS, GLS
52^a^	11.867	Ganoderic acid A	515.3002	C_30_H_44_O_7_	−2.4	561.3062 [M-H+FA]^−^ 497.2923 [M-H-H_2_O]^−^ 453.3016 [M-H-CO_2_-H_2_O]^−^ 435.2914 [M-H-CO_2_-2H_2_O]^−^ 299.1645 [M-H-C_11_H_20_O_4_]^−^ 285.1496 [M-H-C_11_H_18_O_4_-CH_4_]^−^ 195.1013 [M-H-H_2_O-C_19_H_30_O_3_]^−^ 149.0603 [M-H-C_19_H_28_O_3_-H_2_O-CO_2_]^−^	BGLS, RGLS, GLS
53^a^	12.035	Ganolucidic acid B	501.3210	C_30_H_46_O_6_	−2.3	547.3264 [M-H+FA]^−^ 483.3123 [M-H-H_2_O]^−^ 439.3244 [M-H-CO_2_-H_2_O]^−^ 287.2006 [M-H-C_11_H_18_O_4_]^−^ 151.1129 [M-H-H_2_O-CO_2_-C_19_H_28_O_2_]^−^	BGLS, RGLS
54^a^	12.144	Ganoderenic acid D	511.2683	C_30_H_40_O_7_	−3.6	511.2719 [M-H]^−^ 493.2601 [M-H-H_2_O]^−^	BGLS, RGLS
						467.2979 [M-H-CO_2_]^−^ 449.2708 [M-H-CO_2_-H_2_O]^−^ 437.2340 [M-H-CO_2_-2CH_3_]^−^ 299.1641 [M-H-C_11_H_16_O_4_]^−^ 247.1331 [M-H-C_15_H_20_O_4_]^−^ 149.0594 [M-H-C_15_H_20_O_4_-C_6_H_12_O]^−^	
55^a^	12.152	Ganoderenic acid A	513.2847	C_30_H_42_O_7_	−2.1	559.2901 [M-H+FA]^−^ 513.2890 [M-H]^−^ 495.2760 [M-H-H_2_O]^−^ 451.2892 [M-H-H_2_O-CO_2_]^−^ 301.1804 [M-H-C_11_H_16_O_4_]^−^ 285.1871 [M-H-C_11_H_16_O_4_-CH_4_]^−^	BGLS, RGLS
56	12.265	Applanoxidic acid D	527.2646	C_30_H_40_O_8_	−0.8	527.2646 [M-H]^−^ 509.2557 [M-H-H_2_O]^−^ 479.2087 [M-H-H_2_O-2CH_3_]^−^ 465.2651 [M-H-H_2_O-CO_2_]^−^ 435.2183 [M-H-H_2_O-CO_2_-2CH_3_]^−^ 301.1434 [M-H-C_11_H_14_O_4_-CH_4_]^−^	BGLS, RGLS
57	12.308	Lucidenic acid F	455.2430	C_27_H_36_O_6_	−2.0	455.2449 [M-H]^−^ 395.2256 [M-H-CO_2_-CH_4_]^−^ 383.2220 [M-H-C_3_H_4_O_2_]^−^ 351.2325 [M-H-C_3_H_4_O_2_-2CH_4_]^−^ 335.1995 [M-H-C_3_H_4_O_2_-3CH_4_]^−^ 149.0622 [M-H-C_6_H_10_O-C_12_H_16_O_3_]^−^ 249.1497 [M-H-C_12_H_14_O_3_]^−^	BGLS, RGLS
58	12.366	Ganoderic acid LM2	513.2845	C_30_H_42_O_7_	−2.5	513.2892 [M-H]^−^ 495.2773 [M-H-H_2_O]^−^ 480.2482 [M-H-H_2_O-CH_3_]^−^ 436.2629 [M-H-H_2_O-CH_3_-CO_2_]^−^ 421.2420 [M-H-H_2_O-CO_2_-2CH_3_]^−^ 301.1774 [M-H-C_11_H_16_O_4_]^−^ 249.1492 [M-H-C_15_H_20_O_4_]^−^	BGLS, RGLS
59	12.666	Ganoderic acid M	529.2801	C_30_H_42_O_8_	2.6	511.2708 [M-H-H_2_O]^−^ 467.2810 [M-H-H_2_O-CO_2_]^−^ 449.2709 [M-H-CO_2_-2H_2_O]^−^ 434.2467 [M-H-CO_2_-2H_2_O-CH_3_]^−^ 301.1803 [M-H-C_11_H_14_O_4_-H_2_O]^−^ 299.1652 [M-H-C_11_H_16_O_4_-H_2_O]^−^ 263.1283 [M-H-C15H22O4]^−^	BGLS, RGLS, GLS
60^a^	12.757	Lucidenic acid A	457.2587	C_27_H_38_O_6_	−1.9	503.2643 [M-H+FA]^−^ 457.2612 [M-H]^−^ 439.2497 [M-H-H_2_O]^−^ 247.1330 [M-H-C_12_H_18_O_3_]^−^ 209.1171 [M-H-C_15_H_20_O_3_]^−^ 149.0603 [M-H-C_17_H_24_O_5_]^−^	BGLS, RGLS
61	12.813	Ganolucidic acid D	499.3055	C_30_H_44_O_6_	−2.0	545.3107 [M-H+FA]^−^ 499.3078 [M-H]^−^ 481.2959 [M-H-H_2_O]^−^ 437.3059 [M-H-H_2_O-CO_2_]^−^ 287.2020 [M-H-C_11_H_16_O_4_]^−^ 285.1864 [M-H-C_11_H_18_O_4_]^−^	BGLS, RGLS
62	12.936	Ganoderlactone B	453.2274	C_27_H_34_O_6_	−1.9	499.2337 [M-H+FA]^−^ 453.2309 [M-H]^−^ 438.2052 [M-H-CH_4_]^−^ 409.2390 [M-H-CO_2_]^−^ 381.2063 [M-H-C_3_H_4_O_2_]^−^ 379.1918 [M-H-C_3_H_6_O_2_]^−^ 301.1799 [M-H-C_8_H_8_O_3_]^−^ 299.1704 [M-H-C_8_H_10_O_3_]^−^	BGLS, RGLS
63^a^	13.028	Ganoderic acid B	515.3005	C_30_H_44_O_7_	−1.8	561.3039 [M-H+FA]^−^ 515.3029 [M-H]^−^ 497.2922 [M-H-H_2_O]^−^ 453.2987 [M-H-H_2_O-CO_2_]^−^ 301.1807 [M-H-C_11_H_18_O_4_]^−^ 285.1852 [M-H-C_11_H_18_O_4_-CH_4_]^−^	BGLS, RGLS
64	13.122	iso-lucidenic acid E2	515.2642	C_29_H_40_O_8_	−1.6	515.3018 [M-H]^−^ 497.2562 [M-H-H_2_O]^−^ 473.2550 [M-H-C_2_H_2_O]^−^ 455.2452 [M-H-C_2_H_2_O-H_2_O]^−^ 437.2361 [M-H-C_2_H_2_O-2H_2_O]^−^ 425.1971 [M-H-C_2_H_2_O-H_2_O-2CH_3_]^−^ 407.1851 [M-H-C_2_H_2_O-H_2_O-2CH_3_-H_2_O]^−^ 383.2283 [M-H-C_5_H_8_O_2_-2CH_3_]^−^ 301.1812 [M-H-C_2_H_4_O_2_-C_8_H_10_O_3_]^−^	BGLS, RGLS
65	13.362	Ganoderenic acid G	511.2693	C_30_H_40_O_7_	−1.6	557.2750 [M-H+FA]^−^ 493.2586 [M-H-H_2_O]^−^ 449.2609 [M-H-CO_2_-H_2_O]^−^ 434.2480 [M-H-CO_2_-H_2_O-CH_3_]^−^ 419.2230 [M-H-CO_2_-H_2_O-2CH_3_]^−^ 301.1805 [M-H-C_11_H_14_O_4_]^−^ 285.1849 [M-H-C_11_H_14_O_4_-CH_4_]^−^	BGLS, RGLS, GLS
66	13.886	Lucidenic acid F	455.2433	C_27_H_36_O_6_	−1.3	455.2469 [M-H]^−^ 437.2378 [M-H-H_2_O]^−^ 381.2074 [M-H-CO_2_-2CH_3_]^−^ 301.1815 [M-H-C_8_H_10_O_3_]^−^ 299.1633 [M-H-C_8_H_12_O_3_]^−^ 247.1345 [M-H-C_12_H_16_O_3_]^−^ 149.0581 [M-H-C_12_H_16_O_3_-C_6_H_10_O]^−^ 163.1142 [M-H-C_15_H_20_O_3_-CO_2_]^−^	BGLS, RGLS
67^a^	13.976	Ganoderic acid D	513.2850	C_30_H_42_O_7_	−1.5	559.2907 [M-H+FA]^−^ 495.2765 [M-H-H_2_O]^−^ 451.2872 [M-H-H_2_O-CO_2_]^−^ 436.2629 [M-H-H_2_O-CO_2_-CH_3_]^−^ 418.2532 [M-H-2H_2_O-CO_2_-CH_3_]^−^ 301.1810 [M-H-C_11_H_16_O_4_]^−^ 285.1859 [M-H-C_11_H_16_O_4_-CH_4_]^−^ 283.1708 [M-H-C_11_H_18_O_4_-CH_4_]^−^ 247.1337 [M-H-C_15_H_22_O_4_]^−^ 149.0608 [M-H-C_15_H_22_O_4_-C_6_H_19_O]^−^	BGLS, RGLS, GLS
68^a^	14.307	Ganoderenic acid F	509.2534	C_30_H_38_O_7_	−2.1	509.2579 [M-H]^−^ 491.2475 [M-H-H_2_O]^−^ 465.2675 [M-H-CO_2_]^−^ 461.1993 [M-H-H_2_O-2CH_3_]^−^ 447.2560 [M-H-CO_2_-CH_3_]^−^ 417.2074 [M-H-H_2_O-CO_2_-2CH_3_]^−^ 299.1648 [M-H-C_11_H_14_O_4_]^−^	BGLS, RGLS
69	14.534	Ganoleuconin F	569.2750	C_32_H_42_O_9_	−1.1	615.2811 [M-H+FA]^−^ 551.2643 [M-H-H_2_O]^−^ 536.2404 [M-H-H_2_O-CH_3_]^−^ 509.2544 [M-H-C_2_H_4_O_2_]^−^ 491.2463 [M-H-H_2_O-C_2_H_4_O_2_]^−^ 465.2649 [M-H-C_2_H_4_O_2_-CO_2_]^−^ 447.2549 [M-H-H_2_O-C_2_H_4_O_2_-CO_2_]^−^	BGLS, RGLS, GLS
70	14.56	Lucidenic acid D2	513.2487	C_29_H_38_O_8_	−1.3	513.2506 [M-H]^−^ 495.2753 [M-H-H_2_O]^−^ 471.2399 [M-H-C_2_H_2_O]^−^ 441.1919 [M-H-C_3_H_4_O_2_]^−^	BGLS, RGLS, GLS
71	14.752	Ganoderic acid E	511.2694	C_30_H_40_O_7_	−1.4	493.2614 [M-H-H_2_O]^−^ 449.2714 [M-H-H_2_O-CO_2_]^−^ 434.2464 [M-H-H_2_O-CO_2_-CH_3_]^−^ 419.2240 [M-H-H_2_O-CO_2_-2CH_3_]^−^ 301.1803 [M-H-C_11_H_14_O_4_]^−^ 299.1648 [M-H-C_11_H_16_O_4_]^−^ 285.1490 [M-H-C_11_H_14_O_4_-CH_4_]^−^	BGLS, RGLS, GLS
72	14.899	Ganolucidate F	501.3210	C_30_H_46_O_6_	−2.3	501.3225 [M-H]^−^ 457.3336 [M-H-CO_2_]^−^ 303.1987 [M-H-C_10_H_12_O_3_-H_2_O]^−^ 301.1797 [M-H-C_10_H_14_O_3_-H_2_O]^−^ 287.1638 [M-H-C_10_H_12_O_3_-H_2_O-CH_4_]^−^	BGLS, RGLS
73	14.9	Unknown	569.2745	C_32_H_42_O_9_	−1.9	569.2795 [M-H]^−^ 551.2686 [M-H-H_2_O]^−^ 523.3064 [M-H-CH_3_CH_2_OH]- 509.2563 [M-H-C_2_H_4_O_2_]^−^	RGLS
74	14.93	ganoderenic acid K	571.2902	C_32_H_44_O_9_	−1.9	553.2833 [M-H-H_2_O]^−^ 509.2928 [M-H-H_2_O-CO_2_]^−^ 467.2829 [M-H-H_2_O-CO_2_-C_2_H_2_O]^−^ 449.2718 [M-H-2H_2_O-CO_2_-C_2_H_4_O]^−^ 301.1809 [M-H-C_11_H_14_O_4_-C_2_H_4_O_2_]^−^ 299.1646 [M-H-C_11_H_16_O_4_-C_2_H_4_O_2_]^−^ 263.1278 [M-H-C_11_H_16_O_4_-C_2_H_4_O_2_-2H_2_O]^−^	BGLS, RGLS
75	15.072	12β-acetoxy-7β-hydroxy-3,11,15,23-tetraoxo-5α-lanosta-8,20(22)-dien-26-oic acid	569.2763	C_32_H_42_O_9_	1.2	1047.5907 [2M-H]^−^ 569.2277 [M-H]^−^ 551.2683 [M-H-H_2_O]^−^ 509.2571 [M-H-C_2_H_4_O_2_]^−^ 465.2650 [M-H-C_2_H_4_O_2_-CO_2_]^−^ 447.2547 [M-H-H_2_O-C_2_H_4_O_2_-CO_2_]^−^ 429.2469 [M-H-2H_2_O-C_2_H_4_O_2_-CO_2_]^−^	BGLS, RGLS
76	15.197	Unknown	489.2849	C_28_H_42_O_7_	−1.8	443.2807 [M-H-CH_3_CH_2_OH]^−^ 399.2907 [M-H-CH_3_CH_2_OH-CO_2_]^−^ 381.2789 [M-H-CH_3_CH_2_OH-CO_2_-H_2_O]^−^ 287.1999 [M-H-CH_3_CH_2_OH-CO_2_-H_2_O-C_7_H_12_O]^−^	BGLS, RGLS
77	15.198	Ganoderenic acid H	511.2690	C_30_H_40_O_7_	−2.2	511.2708 [M-H]^−^ 493.2615 [M-H-H_2_O]^−^ 449.2705 [M-H-H_2_O-CO_2_]^−^ 434.2469 [M-H-H_2_O-CO_2_-CH_3_]^−^ 149.0603 [M-H-H_2_O-CO_2_-C_19_H_24_O_3_]^−^	BGLS, RGLS
78	15.396	Ganoderic acid GS	525.2483	C_30_H_38_O_8_	−2.1	525.2579 [M-H]^−^ 507.2425 [M-H-H_2_O]^−^ 492.2190 [M-H-H_2_O-CH_3_]^−^ 477.1948 [M-H-H_2_O-2CH_3_]^−^ 463.2520 [M-H-H_2_O-CO_2_]^−^ 448.2253 [M-H-H_2_O-CO_2_-CH_3_]^−^ 433.2068 [M-H-H_2_O-CO_2_-2CH_3_]^−^ 315.1605 [M-H-C_11_H_14_O_4_]^−^ 287.1618 [M-H-C_11_H_12_O_4_-2CH_3_]^−^	BGLS, RGLS, GLS
79	15.666	3β-hydroxyganodernoid D	511.2693	C_30_H_40_O_7_	−2.2	499.3063 [M-H]^−^ 481.2996 [M-H-H_2_O]^−^ 437.3062 [M-H-H_2_O-CO_2_]^−^ 399.2520 [M-H-C_6_H_12_O]^−^ 287.2068 [M-H-C_11_H_16_O_3_-CH_3_]^−^ 235.1702 [M-H-C_15_H_22_O_3_-CH_3_]^−^ 99.0448 [M-H-C_24_H_32_O_5_]^−^	BGLS, RGLS
80	15.617	Ganolucidic acid A	499.3054	C_30_H_44_O_6_	−2.2	545.3109 [M-H+FA]^−^ 481.2974 [M-H-H_2_O]^−^ 437.3081 [M-H-CO_2_-H_2_O]^−^ 287.1999 [M-H-C_11_H_14_O_3_]^−^ 285.1858 [M-H-C_11_H_16_O_3_]^−^	BGLS, RGLS
81	15.619	Ganoderic acid R or ganoderic acid Me	545.3107	C_31_H_46_O_8_	−2.4	545.3108 [M-H]^−^ 511.2691 [M-H-CO_2_]^−^	BGLS, RGLS
82	15.666	Ganodernoid D	567.2595	C_32_H_40_O_9_	−0.8	567.2646 [M-H]^−^ 549.2541 [M-H-H_2_O]^−^ 507.2429 [M-H-C_2_H_2_O]^−^ 477.1957 [M-H-C_2_H_2_O-2CH_3_]^−^ 463.2514 [M-H-C_2_H_2_O-CO_2_]^−^ 433.2040 [M-H-C_2_H_2_O-CO_2_-2CH_3_]^−^ 315.1602 [M-H-C_11_H_14_O_4_-C_2_H_2_O]^−^ 300.1366 [M-H-C_11_H_14_O_4_-C_2_H_2_O-CH_3_]^−^	BGLS, RGLS
83	16.095	Ganoderic acid F	569.2753	C_32_H_42_O_9_	−0.5	615.2807 [M-H+FA]^−^ 551.2645 [M-H-H_2_O]^−^ 509.2561 [M-H-C_2_H_4_O_2_]^−^ 479.2089 [M-H-C_2_H_4_O_2_-2CH_3_]^−^ 465.2658 [M-H-C_2_H_4_O_2_-CO_2_]^−^ 447.2544 [M-H-H_2_O-C_2_H_4_O_2_-CO_2_]^−^ 435.2186 [M-H-C_2_H_4_O_2_-CO_2_-2CH_3_]^−^	BGLS, RGLS, GLS
84	16.419	Ganoderic acid β	499.3055	C_30_H_44_O_6_	−2.0	499.3101 [M-H]^−^ 455.3188 [M-H-CO_2_]^−^ 425.2707 [M-H-CO_2_-2CH_3_]^−^ 287.2024 [M-H-C_11_H_16_O_3_-CH_3_]^−^ 285.1900 [M-H-C_11_H_18_O_3_-CH_3_]^−^ 249.1466 [M-H-C_15_H_22_O_3_]^−^	BGLS, RGLS
85	16.499	Ganoderic acid AM1	513.2850	C_30_H_42_O_7_	−1.5	559.2908 [M-H+FA]^−^ 495.2761 [M-H-H_2_O]^−^ 451.2851 [M-H-H_2_O-CO_2_]^−^ 421.2374 [M-H-H_2_O-CO_2_-2CH_3_]^−^ 399.2550 [M-H-3H_2_O-CO_2_-CH_4_]^−^ 301.1794 [M-H-C_11_H_16_O_4_]^−^ 285.1496 [M-H-C_11_H_16_O_4_-CH_4_]^−^	BGLS, RGLS, GLS
86	16.845	Ganohainanic acid C	499.3054	C_30_H_44_O_6_	−2.2	499.3063 [M-H]^−^ 481.2996 [M-H-H_2_O]^−^ 437.3062 [M-H-H_2_O-CO_2_]^−^ 399.2520 [M-H-C_6_H_12_O]^−^ 287.2068 [M-H-C_11_H_16_O_3_-CH_3_]^−^ 235.1702 [M-H-C_15_H_22_O_3_-CH_3_]^−^ 99.0448 [M-H-C_24_H_32_O_5_]^−^	BGLS, RGLS
87	17.456	Unknown	501.3208	C_30_H_46_O_6_	−2.7	547.3265 [M-H+FA]^−^ 501.3250 [M-H]^−^ 303.2011 [M-H-CO_2_-C_9_H_12_O-H_2_O]^−^ 287.2019 [M-H-C_11_H_18_O_4_]^−^	BGLS, RGLS
88	18.435	Ganorbiformin A	543.3318	C_32_H_48_O_7_	−1.7	543.3336 [M-H]^−^ 501.3252 [M-H-C_2_H_2_O]^−^ 497.2946 [M-H-CH_2_O_2_]^−^ 483.3175 [M-H-C_2_H_4_O_2_]^−^ 457.3330 [M-H-C_2_H_2_O-CO_2_]^−^ 439.3218 [M-H-C_2_H_4_O_2_-CO_2_]^−^ 301.1783 [M-H-C_12_H_18_O_4_-H_2_O]^−^ 287.1630 [M-H-C_12_H_16_O_4_-H_2_O-CH_3_]^−^	BGLS, RGLS
89	18.514	Applanoxidic acid H	529.2798	C_30_H_42_O_8_	−1.7	511.2716 [M-H-H_2_O]^−^ 481.2229 [M-H-H_2_O-2CH_3_]^−^ 467.2844 [M-H-H_2_O-CO_2_]^−^ 437.2354 [M-H-H_2_O-CO_2_-2CH_3_]^−^ 345.1632 [M-H-C_10_H_16_O_3_]^−^ 303.1682 [M-H-H-C_11_H_14_O_5_]^−^	RGLS
90	18.62	iso-ganodernoid D	567.2594	C_32_H_40_O_9_	−1.0	567.2621 [M-H]^−^ 549.2511 [M-H-H_2_O]^−^ 525.2525 [M-H-C_2_H_2_O]^−^ 507.2407 [M-H-C_2_H_2_O_2_-H_2_O]^−^ 495.2039 [M-H-C_2_H_2_O-2CH_3_]^−^ 492.2165 [M-H-C_2_H_4_O_2_-CH_3_]^−^ 477.1937 [M-H-H_2_O-CO_2_-2CH_3_]^−^ 463.2489 [M-H-C_2_H_4_O_2_-CO_2_]^−^	RGLS
91	18.934	Ganoderic acid J	513.2851	C_30_H_42_O_7_	−1.3	513.2865 [M-H]^−^ 495.2778 [M-H-H_2_O]^−^ 451.2870 [M-H-H_2_O-CO_2_]^−^ 433.2751 [M-H-2H_2_O-CO_2_]^−^ 421.2380 [M-H-H_2_O-CO_2_-2CH_3_]^−^ 301.1808 [M-H-C_11_H_16_O_4_]^−^	BGLS, RGLS
92	19.274	Ganoderic acid GS-2	499.3054	C_30_H_44_O_6_	−2.2	499.3066 [M-H]^−^ 455.3153 [M-H-CO_2_]^−^ 437.3089 [M-H-CO_2_-H_2_O]^−^ 357.2432 [M-H-C_8_H_14_O_2_]^−^ 301.1801 [M-H-C_11_H_18_O_3_]^−^ 285.1481 [M-H-C_11_H_18_O_3_-CH_3_]^−^	BGLS, RGLS
93	20.901	Ganohainanic acid C	499.3052	C_30_H_44_O_6_	−2.6	499.3104 [M-H]^−^ 481.2896 [M-H-H_2_O]^−^ 437.3014 [M-H-H_2_O-CO_2_]^−^ 301.1772 [M-H-C_11_H_18_O_3_]^−^ 287.1916 [M-H-C_11_H_16_O_3_-CH_3_]^−^ 285.1847 [M-H-C_11_H_18_O_3_-CH_3_]^−^	BGLS, RGLS
94	20.947	Unknown	543.3308	C_32_H_48_O_7_	−3.5	497.2937 [M-H-CH_2_O_2_]^−^ 479.2817 [M-H-CH_2_O_2_-H_2_O]^−^ 435.2911 [M-H-CH_2_O_2_-H_2_O-CO_2_]^−^	BGLS, RGLS
95	21.202	Ganoderic acid K	573.3069	C_32_H_46_O_9_	0.0	555.2992 [M-H-H_2_O]^−^ 511.3804 [M-H-CO_2_-H_2_O]^−^ 493.2991 [M-H-CO_2_-2H_2_O]^−^ 343.1910 [M-H-C_2_H_4_O_2_-C_9_H_14_O_3_]^−^ 249.1474 [M-H-C_11_H_14_O_4_-C_2_H_4_O_2_-3H_2_O]^−^	RGLS
96	22.699	11-ketodiacetyltangulinsaeure	615.3174	C_34_H_48_O_10_	−0.1	615.3174 [M-H]^−^ 597.3121 [M-H-H_2_O]^−^ 555.2924 [M-H-CH_3_COOH]^−^ 553.3118 [M-H-H_2_O-CO_2_]^−^ 538.3100 [M-H-H_2_O-CO_2_-CH_3_]^−^ 511.3096 [M-H-H_2_O-CO_2_-C_2_H_2_O]^−^ 493.3029 [M-H-2H_2_O-CO_2_-C_2_H_2_O]^−^ 478.2686 [M-H-2H_2_O-CO_2_-C_2_H_2_O-CH_3_]^−^ 344.1960 [M-H-2H_2_O-CO_2_-C_2_H_2_O-CH_3_-C_10_H_14_]^−^ 307.1508 [M-H-2H_2_O-CO_2_-C_2_H_2_O-C_14_H_18_]^−^	RGLS
97	22.879	iso-applanoxidic acid C	525.2484	C_30_H_38_O_8_	−1.9	525.2552 [M-H]^−^ 507.2400 [M-H-H_2_O]^−^ 495.2078 [M-H-2CH_3_]^−^ 477.1963 [M-H-H_2_O-2CH_3_]^−^ 463.2500 [M-H-H_2_O-CO_2_]^−^ 328.1327 [M-H-C_10_H_13_O_4_]^−^	RGLS
98	23.227	Ganolucidic acid E	483.3106	C_30_H_44_O_5_	−2.1	529.3160 [M-H+FA]^−^ 439.3231 [M-H-CO_2_]^−^ 421.3100 [M-H-CO_2_-H_2_O]^−^ 287.2013 [M-H-C_11_H_16_O_3_]^−^ 285.1849 [M-H-C_11_H_18_O_3_]^−^	BGLS, RGLS
99	23.255	Applanoxidic acid A or applanoxidic acid E	511.2684	C_30_H_40_O_7_	8.1	493.2616 [M-H-H_2_O]^−^ 467.2727 [M-H-CO_2_]^−^ 449.2715 [M-H-CO_2_-H_2_O]^−^ 437.2241 [M-H-C_3_H_6_O_2_]^−^ 431.2602 [M-H-CO_2_-2H_2_O]^−^ 419.2223 [M-H-C_3_H_6_O_2_-H_2_O]^−^ 405.2786 [M-H-C_4_H_8_O_2_-H_2_O]^−^ 301.1820 [M-H-C_11_H_14_O_4_]^−^ 299.1609 [M-H-C_11_H_16_O_4_]^−^ 285.1435 [M-H-C_11_H_14_O_4_-CH_4_]^−^	BGLS, RGLS
100	24.006	11β-hydroxy-3,7-dioxo-5α-lanosta-8,24(E)-dien-26-oic acid	483.3104	C_30_H_44_O_5_	−2.5	483.3124 [M-H]^−^ 385.2429 [M-H-C_6_H_10_O]^−^ 345.2072 [M-H-C_9_H_14_O]^−^ 271.1679 [M-H-C_9_H_14_O-2CH_3_-CO_2_]^−^	BGLS, RGLS
101	25.381	Ganoderic acid V	527.3370	C_32_H_48_O_6_	−1.5	527.3405 [M-H]^−^ 485.3237 [M-H-C_2_H_4_O]^−^ 441.3395 [M-H-C_2_H_4_O-CO_2_]^−^ 289.2149 [M-H-C_13_H_18_O_4_]^−^ 195.1011 [M-H-C_19_H_28_O_2_-C_2_H_4_O]^−^	BGLS, RGLS
102	29.09	Unknown	571.3274	C_33_H_48_O_8_	−0.4	525.3240 [M-H-C_2_H_2_O]^−^ 465.3041 [M-H-H_2_O-C_2_H_4_O_2_-CH_2_O]^−^ 315.0470 [M-H-C_11_H_16_O_4_-C_2_H_4_O]^−^ 255.2289 [M-H-C_3_H_6_O_2_-C_4_H_8_O_2_-C_8_H_10_O_3_]^−^ 241.0100 [M-H-C_3_H_6_O_2_-CH_4_-C_4_H_6_O_2_-C_8_H_10_O_3_]^−^ 153.0000 [M-H-C_22_H_26_O_8_]- 99.0450 [M-H-C_26_H_32_O_8_]-	BGLS, RGLS
103	29.37	Unknown	525.3213	C_32_H_46_O_6_	−1.6	571.3267 [M-H+FA]^−^ 483.3126 [M-H-C_2_H_2_O]^−^ 439.3239 [M-H-C_2_H_2_O-CO_2_]^−^ 421.3092 [M-H-C_2_H_2_O-CO_2_-H_2_O]^−^ 287.1987 [M-H-C_12_H_16_O_4_-CH_3_]^−^ 285.1877 [M-H-C_12_H_18_O_4_-CH_3_]^−^	BGLS, RGLS
104	30.354	Ganodernoid G	571.2886	C_32_H_44_O_9_	−4.7	525.3254 [M-H-C_2_H_2_O]^−^ 465.3020 [M-H-C_2_H_4_O_2_-CO_2_]^−^ 315.0486 [M-H-C_11_H_16_O_4_-C_2_H_4_O]^−^ 255.2327 [M-H-C_11_H_12_O_4_-C_2_H_4_O_2_-2CH_4_-H_2_O]^−^ 241.0113 [M-H-C_11_H_12_O_4_-C_2_H_4_O_2_-2CH_4_-2H_2_O]^−^	BGLS, RGLS
105^a^	32.552	Ganoderic acid DM	467.3153	C_30_H_44_O_4_	−3.0	467.3176 [M-H]^−^	BGLS, RGLS
106	34.013	Ganoderic acid TR	467.3159	C_30_H_44_O_4_	−1.7	467.3178 [M-H]^−^ 449.3060 [M-H-H_2_O]^−^ 405.3153 [M-H-H_2_O-CO_2_]^−^ 389.2859 [M-H-H_2_O-CO_2_-CH_3_]^−^ 295.2066 [M-H-CO_2_-2CH_3_-C_6_H_10_O]^−^	BGLS, RGLS
107	36.159	Unknown	475.3058	C_28_H_44_O_6_	−1.5	475.3071 [M-H]- 457.2947 [M-H-H_2_O]- 431.3174 [M-H-CO_2_]- 413.3052 [M-H-CO_2_-H_2_O]-	BGLS, RGLS
108	38.022	Unknown	475.3056	C_28_H_44_O_6_	−1.9	475.3081 [M-H]- 431.3163 [M-H-CO_2_]- 413.3079 [M-H-CO_2_-H_2_O]-	BGLS, RGLS
109	38.176	Linoleic acid	279.2330	C_18_H_32_O_2_	−1.6	279.2337 [M-H]^−^	BGLS, RGLS, GLS

a Identified with reference compounds.

*GLS, Ganoderma lucidum spore; BGLS, sporoderm-broken Ganoderma lucidum spore; RGLS, sporoderm-removed Ganoderma lucidum spore*.

### Zebrafish Assays to Assess Immunomodulatory Activities

**Figure 4:**
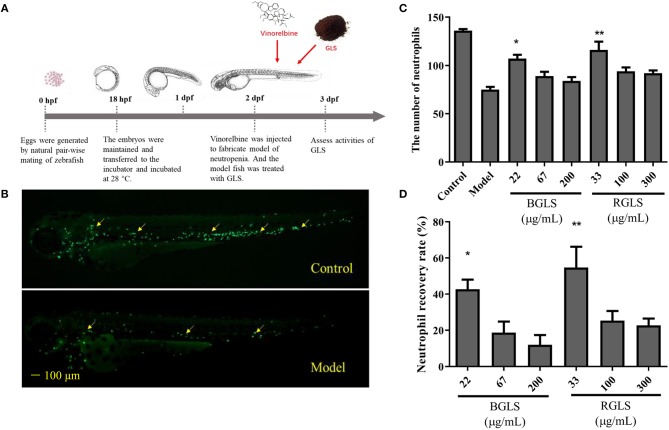
Assessment of immunoactivity of *Ganoderma lucidum* spores on zebrafish models of neutropenia. **(A)** Scheme of zebrafish husbandry and treatment. **(B)** Representative fluorescent images of *Tg (mpx:GFP)* transgenic larvae of control and model groups. **(C)** Neutrophils count in zebrafish of different groups. **(D)** Neutrophil recovery rate (%) of different groups. BGLS, sporoderm-broken *Ganoderma lucidum* spores; RGLS, sporoderm-removed *Ganoderma lucidum* spores. Data are expressed as mean ± SEM, *n* = 10. *P < 0.05, **P < 0.01 vs. Model.

**Figure 5:**
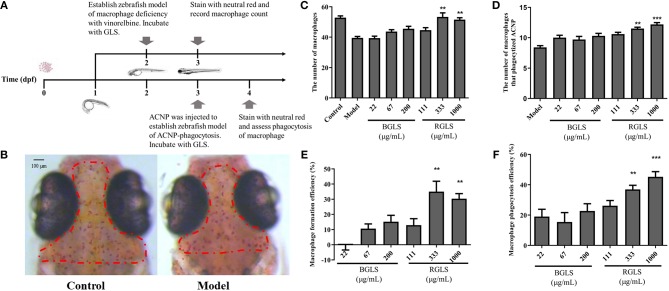
Assessment of immunoactivity of *Ganoderma lucidum* spores on zebrafish models of macrophage deficiency and macrophage phagocytosis. **(A)** Overview of zebrafish development and experimental protocol. **(B)** Represent images of macrophages in healthy fish and zebrafish model of macrophage deficiency. **(C)** Macrophages count in zebrafish of different groups. **(D)** The number of macrophages that phagocytize active carbon nanoparticles of different groups. **(E)** Macrophage formation efficiency (%) of different groups. **(F)** Macrophage phagocytosis efficiency (%) of different groups. BGLS, sporoderm-broken *Ganoderma lucidum* spores; RGLS, sporoderm-removed *Ganoderma lucidum* spores. Data are expressed as mean ± SEM, *n* = 10. **P < 0.01, ***P < 0.001 vs. Model.

The large difference of chemical profiles of BGLS and RGLS indicated their bioactivities might vary. Due to its convenience and optical accessibility, the zebrafish (*D. rerio*) has been widely adopted as a model for understanding the mechanisms of development and recently, there has been increasing use of the this organism in varied fields including immunology (Novoa and Figueras, 2012). Therefore, as GLS is considered as a potential immunotherapy agent (Cao et al., 2018), the zebrafish models of neutropenia and macrophage deficiency were employed to evaluate the immunomodulatory activities in terms of neutropenia recovery, macrophage formation, and macrophage phagocytosis. Vinorelbine was intravenously injected at 1 and 0.25 ng per larva to generate the neutropenia and macrophage deficiency models, respectively (Figures 4A, 5A). As shown in Figure 4B, the fluorescence intensity of the model group decreased significantly compared with the control, indicating the model was successfully established. After exposing to different concentrations of BGLS and RGLS for 24 h, the number of neutrophils in the larvae recovered with different degrees. BGLS of 22 μg/mL and RGLS of 33 μg/mL significantly improved the neutrophils compared with the model (*P* < 0.05 or 0.01), while RGLS exhibited more potent effects than BGLS (Figures 4C,D).

In the macrophage deficiency model created by vinorelbine (Figure 5B), however, only RGLS of higher concentrations (i.e., 333 and 1,000 μg/mL) could significantly promote the formation of macrophages compared with the model (*P* < 0.01), while BGLS showed very weak effects (Figures 5C,D). Likewise, in the macrophage phagocytosis model, RGLS of 333 and 1,000 μg/mL significantly increased the number of macrophages that phagocytized ACNP, while BGLS of tested concentrations exhibited slight improvement (Figures 5E,F).

### Screening Active Compounds by PLSR-Based Activity Ranking

The above results suggested the immunomodulatory activity of GLS was positively correlated with the content of triterpenoids. However, more than 100 triterpenoids were detected in the GLS, and their contents varied considerably, making it difficult to assign the activity to a single molecule. In addition, as most of these triterpenoids were not commercially available, it would be rather labor-intensive and costly to purify and test the compounds individually. To rapidly screen potential active compounds, we employed the partial least squares regression (PLSR) algorithm to find the correlation between the peak area information (X variables) and the activity (Y variable, i.e., neutrophil recovery rate, macrophage formation efficiency or macrophage phagocytosis efficiency). The activity index was proposed to evaluate the contribution of each constituent to the activities, which was calculated using the following formula:

$$\begin{array}{l} \text{Y}=\overset{n=109}{\underset{i=1}{\sum }}a_{i}x_{i}C_{i} \end{array}$$

where Y was the activity of the tested sample; *a*_*i*_ was the activity index of constituent *i*; *x*_*i*_ was the peak area of constituent *i* in the tested sample; *C*_*i*_ was the relative concentration of the tested sample, which was defined as *c*_*i*_*/c*_*max*_, where *c*_*i*_ was the concentration of the tested sample and *c*_*max*_ was the max concentration in each group.

The PLSR model was created according to PLS-W2A (Wegelin, 2000). The dataset obtained was transferred to the center of the multidimensional coordinate system to identify potential active compounds that had high activity index in all the three bioassays. All programs were operated by Spyder with Python 3.6 (Anaconda, Inc., Austin, Texas, USA) software.

**Figure 6:**
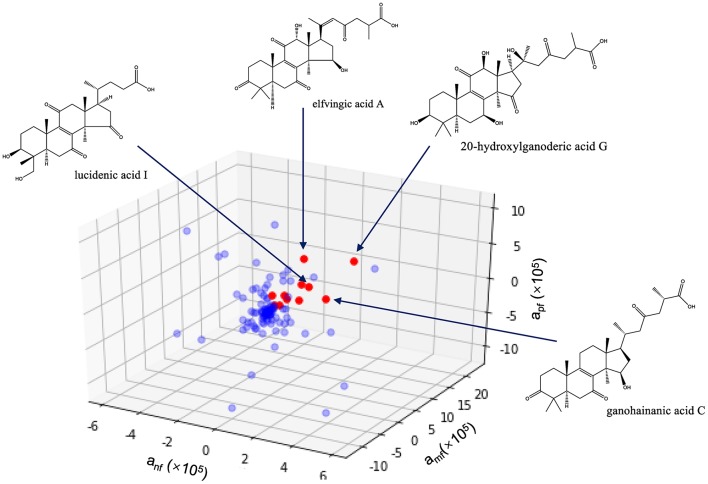
Identification of immunoactive compounds of *Ganoderma lucidum* spore by activity index. anf, amf, apf are the activity indices of the compound, representing its effects on neutrophil recovery, macrophage formation and macrophages phagocytosis, respectively.

As shown in Figure 6, 11 compounds, namely 20-hydroxylganoderic acid G, elfvingic acid A, elfvingic C, lucidenic acid I, ganohainanic acid C, ganoderic acid β, methyl lucidenate E2, dehydrolucidenic acid N, applanoxidic acid G, ganolucidic acid D and ganoderenic acid H were identified as potential immunoactive compounds that ranked high in neutropenia recovery, macrophage formation, and macrophage phagocytosis. However, these candidates needed meticulous validation *in vivo* and *in vitro* to confirm their pharmacological effects.

## Discussion

It has been reported that *G. lucidum* contains over 400 bioactive compounds, including triterpenoids, polysaccharides, nucleotides, sterols, steroids, fatty acids and proteins/peptides, which have various medicinal effects. In the present study, we identified chemical constitutes of GLS by LC-MS and the main constitutes of GLS were identified as triterpenoids. We further employed zebrafish as the animal model to investigate the immunomodulatory activities of GLS, due to its advantages in immune-related research, which included: (1) having complete (innate and adaptative) immune systems, and possessing neutrophils and macrophages in adults and larvae; (2) having a relatively rapid life cycle and was easy to maintain; (3) quickly producing large numbers of offspring that could be assayed in multi-well plates and treated with different chemicals. Finally, activity index was proposed to evaluate the contribution of each constituent to the activity to screen potential immunoactive ones.

In a previous study, Ahmadi and Riazipour revealed that *G. lucidum* could improve macrophage function through cytokine and NO release (Ahmadi and Riazipour, 2007). Besides, several pharmacological studies have shown that *G. lucidum* can play an antitumor role through the regulation of the immune system (Boh et al., 2007). A finding suggested that the triterpenes of *G. lucidum* inhibited anti-lung cancer *in vitro* and *in vivo* via enhancement of immunomodulation and induction of cell apoptosis (Liang et al., 2013). Therefore, it can be predicted that GLS may affect the formation and phagocytic function of macrophages to exert the immunomodulatory effect.

Among the compounds identified in *G. lucidum*, triterpenoids are most widely investigated. Besides the anticancer activities which have been reported by many studies, triterpenoids also showed good immunomodulatory effects. It has been reported that the triterpenoids extract from *G. lucidum* have anti-inflammatory and anti-proliferative effects, which are mediated through the inhibition of NF-κB and AP-1 signaling pathways in macrophages (Dudhgaonkar et al., 2009). As a major triterpene of GL and GLS, previous studies suggested that Ganoderic acid A played a role in immunomodulation through inhibiting the release of proinflammatory mediators like IL-1β, IL-6 and TNF-α (Chi et al., 2018). A study also suggested that Ganoderic acid A-treatment not only enhanced cell-mediated immune responses, but also potentiated antitumor immune responses by activating IFN-γ producing CD8+ T cells (Radwan et al., 2015). Liu et al. found that ganoderic acid C_1_ had an effect on immune system and significantly suppressed murine macrophage TNF-α production, which was associated with suppression of NF-κB (Liu et al., 2015). These studies gave possible direction of our further mechanism study.

## Conclusion

This work demonstrates the feasibility of mass spectrometry molecular networking coupled with zebrafish-based bioassays and chemometrics for active constituent identification of complex herbal medicine. An efficient MS data processing method integrating molecular networking and targeted LC-MS analysis was established to comprehensively profile GLS, leading to the characterization of 109 constituents. Immunomodulatory activities of different GLS samples were evaluated by zebrafish models of neutrophil or macrophage deficiency, in which RGLS showed better therapeutic effects than BGLS. Moreover, a three-dimensional activity index approach based on PLSR was developed to identify active constituents in GLS that were effective on all the three bioassays, which included 20-hydroxylganoderic acid G, elfvingic acid A, elfvingic C, and etc. Future works are needed to validate the pharmacological effects of these compounds using purified substances on various *in vivo* assays. Also possible for further work is employing ions in the molecular network rather than identified peaks for correlation analysis, to explore the potential relationship between bioactivities and certain ions/structures.

## Data Availability Statement

The datasets generated for this study are available on request to the corresponding author.

## Ethics Statement

The animal study was reviewed and approved by the International Association for Assessment and Accreditation of Laboratory Animal Care (AAALAC).

## Author Contributions

ZL, JX, and YW designed the research. ZL, YS, and XZ performed the experimental work and analyzed the data. ZL, JX, and HW prepared the GLP extracts. ZL, YS, LZ, and YW participated in the preparation of manuscript. All authors proofread the paper and approved the final version of the manuscript.

### Conflict of Interest

The authors declare that the research was conducted in the absence of any commercial or financial relationships that could be construed as a potential conflict of interest.
