# Supplementary material for: Screening Immunoactive Compounds of Ganoderma lucidum Spores by Mass Spectrometry Molecular Networking Combined With in vivo Zebrafish Assays
Source: Front Pharmacol. 2020 Mar 18;11:287. doi: 10.3389/fphar.2020.00287 (PMC7093641; doi:10.3389/fphar.2020.00287)
Supplement: Supplementary file 1 [file Data_Sheet_1.pdf]

Supplementary Material

1 Supplementary Methods

Determination of MTCs of BGLS and RGLS on zebrafish of neutropenia

2-dpf *Tg (mpx:GFP)* transgenic zebrafish embryos were distributed into 6-well plates, with 30 larvae in 3 mL system fish water for each well. The model group was given vinorelbine (1 ng per larva) by intravenous microinjection to establish the neutropenia model. In treatment groups, zebrafish of neutropenia were exposed to different concentrations of BGLS (100, 200, 400, 600 µg/mL) or RGLS (300, 400, 500, 600, 800 µg/mL). After administration, the zebrafish were incubated in a 28 °C incubator for 24 h and then MTCs of BGLS or RGLS were determined. BGLS of 100 and 200 µg/mL showed no toxicity on the zebrafish compared with the model group, while BGLS of 400 and 600 µg/mL caused obvious pericardial edema with incidence of 46.7% and 56.7%, respectively. RGLS of 300 µg/mL showed no toxicity on the zebrafish, while RGLS of 400, 500, 600 and 800 µg/mL caused obvious pericardial edema with incidence of 53.3%, 63.3%, 66.7% and 66.7%, respectively. Therefore, MTCs of BGLS and RGLS on the neutropenia model were determined at 200 and 300 µg/mL, respectively.

Determination of MTCs of BGLS and RGLS on *Albino* zebrafish

2-dpf *Albino* zebrafish embryos were distributed into 6-well plates, with 30 larvae in 3 mL system fish water for each well. In treatment groups, zebrafish were exposed to different concentrations of BGLS (100, 200, 300 and 400 µg/mL) or RGLS (250, 500, 1000 and 2000 µg/mL). After administration, the zebrafish were incubated in a 28 °C incubator for 48 h and then MTCs of BGLS or RGLS were determined. BGLS of 100 and 200 µg/mL showed no toxicity on the zebrafish compared with the control group, while BGLS of 300 and 400 µg/mL caused delay in yolk sac absorption with incidence of 13.3% and 100%, respectively. RGLS of 250, 500 and 1000 µg/mL showed no toxicity on the zebrafish, while RGLS of 2000 µg/mL caused obvious delay in yolk sac absorption and bradykinesia, with incidence of 100%. Therefore, MTCs of BGLS and RGLS on *Albino* zebrafish were determined at 200 and 1000 µg/mL, respectively.

.

2 Supplementary Figures and Tables

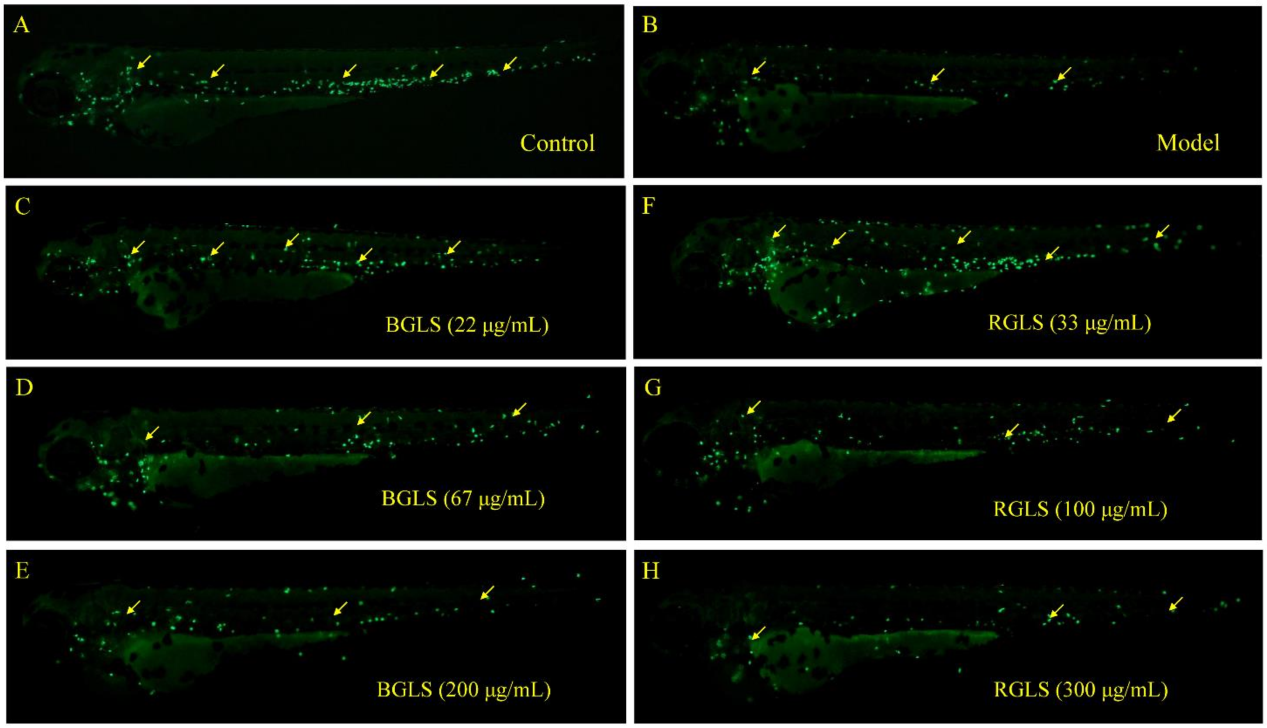

Supplementary Figure 1. Represent fluorescent images of neutrophils in *Tg (mpx:GFP)* transgenic zebrafish of different groups. BGLS, sporoderm-broken *Ganoderma lucidum* spores. RGLS, sporoderm-removed *Ganoderma lucidum* spores.

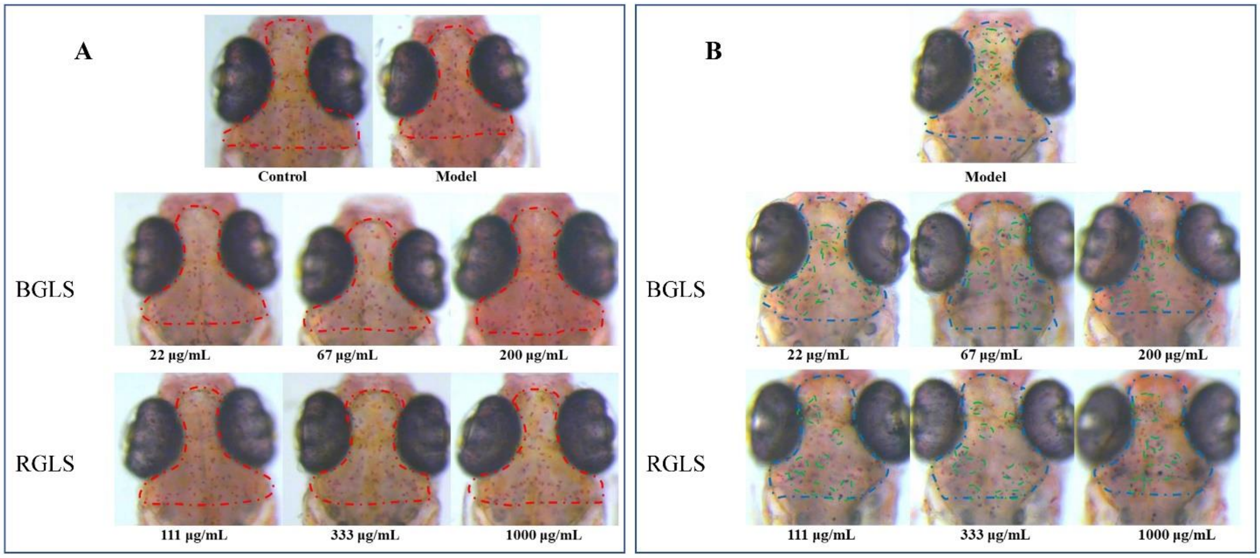

Supplementary Figure 2. Represent images of macrophages *Albino* zebrafish in different groups. (A) Zebrafish models of macrophage deficiency. (B) Zebrafish models of macrophage phagocytosis. BGLS, sporoderm-broken *Ganoderma lucidum* spores. RGLS, sporoderm-removed *Ganoderma lucidum* spores.
